# Preliminary observations of tool-processing behaviour in Hawaiian crows *Corvus hawaiiensis*

**DOI:** 10.1080/19420889.2018.1509637

**Published:** 2018-10-21

**Authors:** Barbara C. Klump, Bryce M. Masuda, James J. H. St Clair, Christian Rutz

**Affiliations:** a Centre for Biological Diversity, School of Biology, University of St Andrews, St Andrews, UK; b Institute for Conservation Research, San Diego Zoo Global, Volcano, Hawaii, USA

**Keywords:** Alala, construction behaviour, corvid, extractive foraging, Hawaiian crow, material selectivity, New Caledonian crow, tool manufacture, tool use

## Abstract

Very few animal species habitually make and use foraging tools. We recently discovered that the Hawaiian crow is a highly skilled, natural tool user. Most captive adults in our experiment spontaneously used sticks to access out-of-reach food from a range of extraction tasks, exhibiting a surprising degree of dexterity. Moreover, many birds modified tools before or during deployment, and some even manufactured tools from raw materials. In this invited addendum article, we describe and discuss these observations in more detail. Our preliminary data, and comparisons with the better-studied New Caledonian crow, suggest that the Hawaiian crow has extensive tool-modification and manufacture abilities. To chart the full extent of the species’ natural tool-making repertoire, we have started conducting dedicated experiments where subjects are given access to suitable raw materials for tool manufacture, but not ready-to-use tools.

Only a handful of bird species habitually make and use foraging tools [1], amongst them the well-known New Caledonian crow *Corvus moneduloides* [2,3]. Recently, we discovered that a second tropical corvid – the Hawaiian crow or ‘Alalā *C. hawaiiensis* – is a highly skilled, natural tool user [4]. The species became extinct in the wild in 2002, but is being propagated in two captive breeding facilities on the islands of Hawai‘i and Maui, to support an ongoing reintroduction programme [5,6]. To test the tool-using abilities of ‘Alalā, we provided subjects (104 of 109 captive birds in 2013) with: a wooden log containing several meat-baited extraction tasks (crevices and drilled holes); vegetation as material for tool manufacture (dead forked branches, and on Hawaii Island, additionally live forked branches and fern sections of native species); and assorted sticks as potential tools (for details, see [4]). Our experiment revealed that tool use is a species-wide capacity (93% of all adult birds spontaneously used tools to extract bait), and that at least some birds are capable of modifying (abbreviated henceforth as MO) and manufacturing (MA) tools [4]. Here, we provide a more detailed description of these preliminary observations.

Shortening sticks (MO-1) was the most frequent tool modification, with 67% of the 64 individually-tested tool users exhibiting this behaviour either before or during tool use; some representative examples are shown in Supplementary Video 2 of our earlier paper [4]. Additionally, 8% of birds performed other modes of modification: nibbling the end of a tool (MO-2; 4 cases by 3 birds); removing parts of a leaf from a stem (MO-3; 1 case); reducing the size of pieces of wood through forceful pecking (MO-4; 3 cases by one bird) or of a piece of bark by subtracting material (MO-5; 1 case; see also bark-tool manufacture discussed below); and stripping bark from a stick (MO-6; 1 case).

Especially interesting is behaviour MO-6, where a bird removed bark from a stick it had previously inserted into a vertical hole in the experimental log (Figure 1a) – a behaviour that resembled the bark stripping expressed by some New Caledonian crows during hooked stick tool manufacture from forked branches [7,8]. Removing the outer layer of bark may enhance tool functionality by reducing friction (a smooth tool may be easier to insert) and/or increasing visibility (a light-coloured tool may be easier to see when deployed in dark holes or crevices) [7]. While our subject used the tool again following bark removal, it appeared to insert the non-processed end (the tool was hidden behind the bird’s body, so tool placement had to be inferred).

Tool manufacture was much rarer than tool modification, with only 14% of all individually-tested tool users making and subsequently using tools from dead and/or live plant materials. This is unsurprising, since suitable stick tools were always readily available as part of the experimental set-up [4]. We observed birds: snipping-off twigs from dead forked branches (MA-1; 3 cases by 3 birds; see Figure 1b); reducing the size of a piece of wood by chiselling, so it could be used as a tool (MA-2; 1 case); and removing: the end of a leaf petiole from a leaf (MA-3; 1 case), most of the leafed section of a live plant stem (MA-4; 1 case), a young plant growing naturally in the aviary, with subsequent processing of the material (MA-5; 1 case), or bark from either the experimental log (MA-6; 10 cases by 2 birds; see Figure 1c) or from larger pieces of bark (MA-7; 2 cases by 2 birds). Examples of behaviours MA-1, MA-2, MA-5 and MA-6 can be found in Supplementary Video 2 of our earlier paper [4].

**Figure 1.**
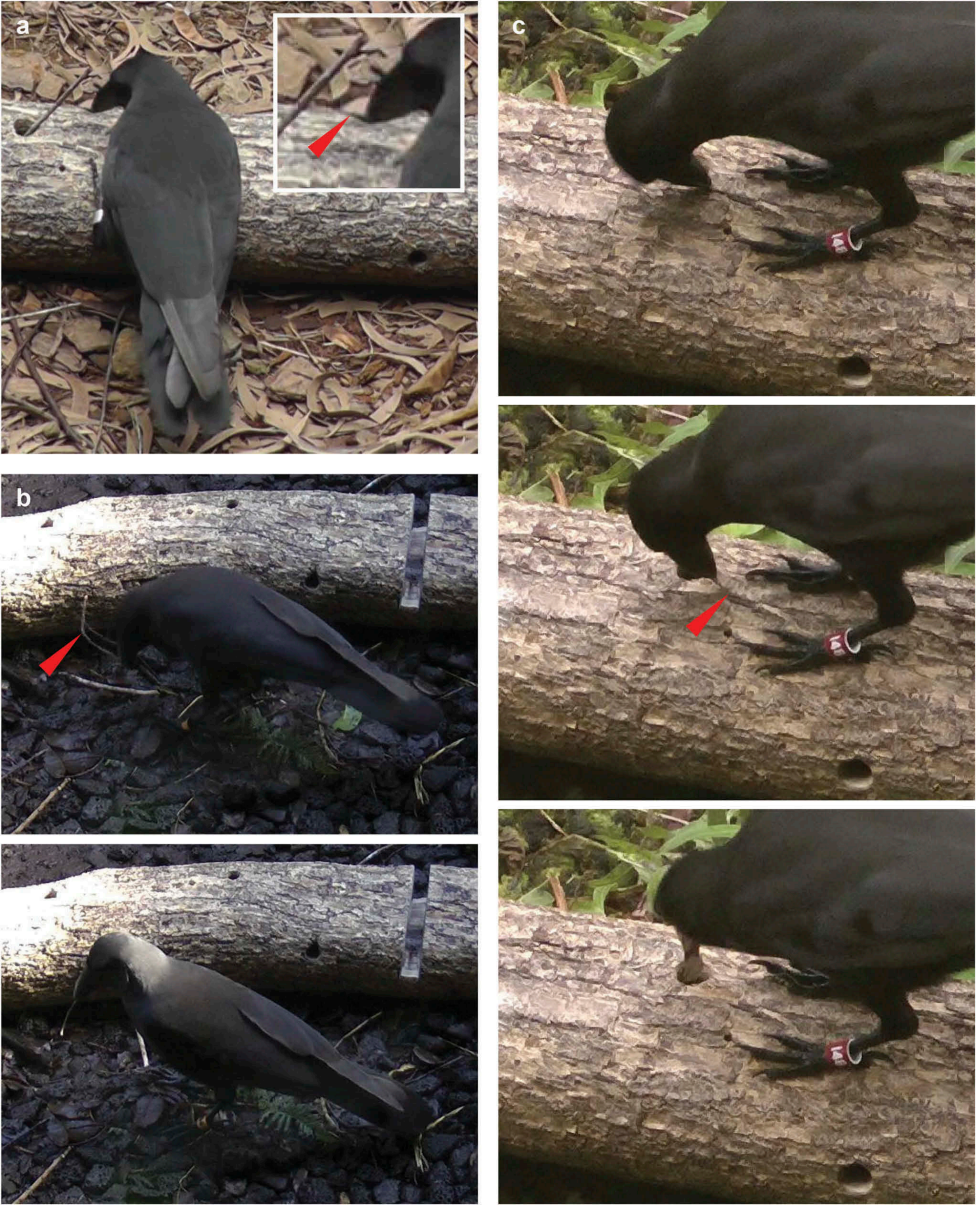
Examples of spontaneous tool-processing behaviours observed during a controlled experiment with captive ‘Alalā ([4]; still images taken from video footage, cropped and resized). (a) Adult female (studbook number #77) removing bark from a stick it had previously inserted into a hole in the experimental log (Koa *Acacia koa*) (1 image plus zoomed-in inset; modification behaviour MO-6). (b) Adult female (#94) snipping-off a twig from a supplied forked branch (2 images; manufacture behaviour MA-1). (c) Adult female (#148) peeling-off bark from the experimental log (3 images; manufacture behaviour MA-6). In all three cases, birds used the modified or manufactured tool for probing into holes or crevices in the experimental log.

The removal of bark to be used as tools (MA-6, MA-7; see Figure 1c) is noteworthy since wild ‘Alalā in the late 1970s and early 1980s spent a large proportion of their foraging time flaking bark to access hidden prey ([9,10]; although no tool use was reported). New Caledonian crows also frequently engage in bark flaking (43% of recorded bill interactions in a study using bird-borne miniature video cameras [11]), and exhibit a wide range of tool-processing behaviours [2,3,8,11–18], but to our knowledge, there have been no reports of bark tool use in the wild. Interestingly, the only observation to date of a wild American crow *C. brachyrhynchos* using a tool for (presumed) extractive foraging involved the use of a wooden splinter, which the bird had released from a fence post through pecking and pulling [19]. A useful non-corvid comparison is provided by nuthatches *Sitta* spp. that are known to use bark tools, illustrating how this tool type can aid foraging for arthropod prey (for a review and photos illustrating the behaviour, see [20]).

The study of ‘Alalā tool behaviour is still in its infancy, and many exciting research opportunities lie ahead [4,21]. Our preliminary observations have confirmed that ‘Alalā are capable of modifying and manufacturing tools in a variety of ways, paving the way for dedicated experiments that systematically chart the species’ natural tool-making repertoire, as well as its ontogenetic development [4,22–24]. Importantly, subjects need to be tested under conditions where they have access to a wide variety of naturalistic foraging tasks (including those routinely encountered by wild New Caledonian crows [2,3,11,12,16]), and different plant materials for tool manufacture, but *not* to sticks or other objects that could be used as tools without further processing (as was the case in our first study [4]). This work programme, which we have recently launched, borrows experimental designs we developed for assaying the tool-manufacture behaviour of wild-caught New Caledonian crows [8,25–27]. With a cohort of ‘Alalā recently released into the wild, our controlled studies in captivity can be complemented with detailed field observations, to determine the extent to which birds use tools, and other extractive foraging techniques, under contemporary ecological conditions [4].
